# Moxibustion for the treatment of diabetic peripheral neuropathy: A systematic review and meta-analysis following PRISMA guidelines

**DOI:** 10.1097/MD.0000000000022286

**Published:** 2020-09-25

**Authors:** Yumeng Tan, Jun Hu, Bing Pang, Lijuan Du, Yanan Yang, Qing Pang, Meizhen Zhang, Qian Wu, Yi Zhang, Qing Ni

**Affiliations:** a Department of Endocrinology; b Department of Cardiovascular, Guang’an Men Hospital, China Academy of Chinese Medical Sciences; c Beijing University of Chinese Medicine, Beijing, China.

**Keywords:** diabetic peripheral neuropathy, meta-analysis, moxibustion

## Abstract

**Background:**

At present, the effect of western-medicine (WM) therapy to treat diabetic peripheral neuropathy (DPN) is limited. Moxibustion is a representative external treatment in traditional Chinese medicine that has been beneficial to DPN. We aim to systematically assess the efficacy and safety of moxibustion in treating DPN, following PRISMA guidelines.

**Methods:**

Eight electronic databases were searched to acquire information on eligible trials published from inception to June 1, 2019. We included randomized controlled trials (RCTs) applying moxibustion therapy with a minimum of 14-days treatment duration for DPN patients compared with placebo, no intervention, or conventional WM interventions. The primary outcomes in our study include the sensory-nerve conduction velocity (SNCV) and motor-nerve conduction velocity (MNCV). We used the Cochrane Collaboration Risk of Bias tool to assess the methodological quality of eligible RCTs. Statistical analyses were conducted using Review Manager 5.3. Risk ratios (RR) and mean differences (MD) were calculated with a 95% confidence interval (CI). The *χ*^2^ test was applied to assess the heterogeneity.

**Results:**

In total, 11 RCTs were included that involved 927 DPN patients. Compared with the control group, there was an increase in median MNCV (MD = 6.26, 95% CI 2.64–9.89, Z = 3.39, *P* = .0007) and peroneal MNCV (MD = 6.45, 95% CI 5.30–7.61, *P* < .00001). There was also an increase in median SNCV (MD = 6.64, 95% CI 3.25–10.03, *P* = .0001) and peroneal SNCV (MD = 3. 57, 95% CI 2.06–5.09, Z = 4.63, *P* < .00001) in the treatment groups. The treatment groups receiving moxibustion therapy indicated a more significant improvement in total effectiveness rate (RR = 0.25, 95% CI 0.18–0.37, Z = 7.16, *P* < .00001). Toronto Clinical Scoring System indicated a significant decrease in the treatment groups (MD = −2.12, 95% CI −2.82 to 1.43, *P* < .00001). Only 1 study reported that treatment groups experienced no adverse reactions. The other 10 studies did not mention adverse events.

**Conclusions:**

Moxibustion therapy may be an effective and safe option for DPN patients but needs to be verified in further rigorous studies.

## Abstract 2

Supplemental Digital Content is available in the text

## 1 Introduction

A recent rapid increase in the number of diabetic patients has made diabetes a serious public-health concern. The latest edition of IDF Diabetes Atlas states that 463 million adults are currently living with diabetes worldwide and estimates that there will be 578 million adults with diabetes by 2030.^[1]^ Peripheral neuropathy is one of the common chronic complications of diabetes mellitus, with the incidence increasing with the escalating number of diabetics. The prevalence of neuropathy in patients with diabetes is approximately 30% with up to 50% eventually developing neuropathy during their disease.^[2]^ The most common presentation of diabetic peripheral neuropathy (DPN) is distal symmetric polyneuropathy that is characterized by pain, numbness, abnormal sensation, and weakness that affect the nerves in distal extremities^[3]^ that can easily lead to conditions such as diabetic foot, foot ulcer, and amputation, adding further burden to the public healthcare. Controlling glycemia and cardiovascular risks are now considered to be vital in the management of DPN patients.^[4]^ There are several measures available for addressing the painful symptoms including mecobalamin, tricyclic compounds, antioxidant alpha-lipoic acid, anticonvulsants, opiates, etc. However, few therapies are available for the improvement of painless symptoms. Moreover, the efficacy of western-medicine (WM) is poor and limited. For example, a study by Su et al^[5]^ pointed out that a simple application of mecobalamin could not improve the ischemia and anoxemia status of nervous tissues.

For this reason, many physicians have started to explore what the traditional Chinese medicine (TCM) could offer to DPN therapy. TCM prevents and cures diseases based on its guidance that includes many therapeutic options (e.g., herbal medicine, Chinese patent medicine, acupuncture, moxibustion, manipulation, etc.). External therapies of TCM are widely used in clinical practice; in particular, acupuncture has been proven to be clinically effective and is being widely applied to treat DPN.^[6,7]^ Besides acupuncture, moxibustion is also a representative external treatment in TCM that can regulate and harmonize qi and blood, warm meridians, and activate blood circulation, and thus that treats and prevents diseases. The clinical effectiveness of moxibustion in treating DPN has been widely recognized. Recent studies showed that moxibustion can increase serum superoxide dismutase concentration,^[8]^ reduce free-radical production, prevent impairments of nerve tissues resulting from free-radical accumulation, and alleviate neuro-inflammation possibly by inhibiting NF-κB and activating Nrf2.^[9]^ The efficacy of moxibustion therapy has been claimed by many studies but there is still a lack of objective evaluation of its benefits in treating DPN. Therefore, the effectiveness of moxibustion therapy in DPN remains controversial and its application is limited.

We conducted this meta-analysis to assess the strength of the current evidence to support the efficacy and safety of moxibustion for the treatment of DPN that might be a novel treatment strategy for DPN.

## 2 Methods

Our systematic review was registered with PROSPERO in June 2019 (registration number CRD 42019138266). The methods of this meta-analysis were performed following the PRISMA guideline.^[10]^

### 2.1 Search strategy

To identify eligible studies, the main search was conducted in the following 8 electronic databases: Cochrane Library, PubMed, EMBASE, Web of Science, Chinese National Knowledge Infrastructure database (CNKI), Chinese Biomedical Database (CBM), Chinese Science and Technique Journal Database (VIP), and Wan Fang Database up to June 1, 2019. The search was performed using various combinations of Medical Subject Headings (MeSH) and non-MeSH terms. The search terms used included diabetic neuropathy, diabetic peripheral neuropathy, diabetic neuropathies, DPN, moxibustion, moxa, moxa-moxibustion, warm-moxibustion, mild-moxibustion, indirect-moxibustion, randomized controlled trial, controlled clinical trial, randomized, placebo and randomly for searching Cochrane Library, PubMed, EMBASE, and Web of Science. Corresponding Chinese terms were used when searching for VIP, CBM, Wan Fang, and CNKI databases. A complete search strategy used for PubMed is shown in Supplementary Material File1.

### 2.2 Study design

#### 2.2.1 Inclusion criteria

1. Study types: randomized controlled trials (RCTs) in English or Chinese were included.
2. Participants: age≥18 years, with nationally or internationally recognized diagnostic criteria of DPN by organizations such as the WHO, American Diabetes Association, 2009 Chinese Medical Doctor Association Guidelines for diagnosis and treatment of diabetic peripheral neuropathy being met by all participants.
3. Interventions: Moxibustion without restrictions on the types of moxibustion, equipment, materials, points, or frequency. The control interventions were no intervention, placebo, or WM therapy. Besides, routine hypoglycemic therapy should be used in both groups.
4. Outcomes: Primary: the sensory-nerve conduction velocity (SNCV) and motor-nerve conduction velocity (MNCV). Secondary: the total effectiveness rate, Toronto Clinical Scoring System (TCSS),^[11]^ glucose indices (e.g., FBG, 2hPG, and HbA1c), and adverse events.

#### 2.2.2 Exclusion criteria

Study types: randomized controlled animal study. Diagnosis: participants with peripheral neuropathy not caused by diabetes. Interventions: moxibustion combined with other TCM treatments (e.g., acupuncture, foot bath, herbal medicine, etc.)

### 2.3 Data extraction

Two reviewers extracted the following data: study ID, sample size, average age, gender, duration of diabetes, diabetes types, TCM-syndrome types, DPN diagnostic criteria, interventions (Types and dosage of WM, a dose of moxibustion therapy), treatment duration, moxibustion treatment times, and outcome measures. Discrepancies were resolved through discussions with a third party (QN).

### 2.4 Risk-of-bias assessment

Based on the Cochrane Risk of Bias Tool,^[12]^ 2 reviewers independently evaluated the methodological quality of the included studies. The following 7 elements were assessed: random-sequence generation, allocation concealment, blinding of participants and personnel, blinding of outcome assessment, incomplete-outcome data, selective reporting, and other bias. Any discrepancies were resolved by consensus.

### 2.5 Data analysis

Statistical analyses were performed using the RevMan 5.3 software. Dichotomous data were expressed as the risk ratio (RR), and continuous outcomes between groups as mean difference (MD), both with a 95% confidence interval (CI).

We accessed heterogeneity by the *χ*^2^ test. When there was substantial heterogeneity (*P* < .10, I^2^>50%), we used the random effects model to analyze the data. Otherwise, a fixed-effect model was applied (i.e., when *P* > .1 or I^2^ < 50%).^[13]^ The sensitivity analysis and subgroup analysis would be performed to explore the possible sources of heterogeneity. Furthermore, publication bias was assessed using a funnel plot.^[14]^

## 3 Results

### 3.1 Searching result

**Figure 1:**
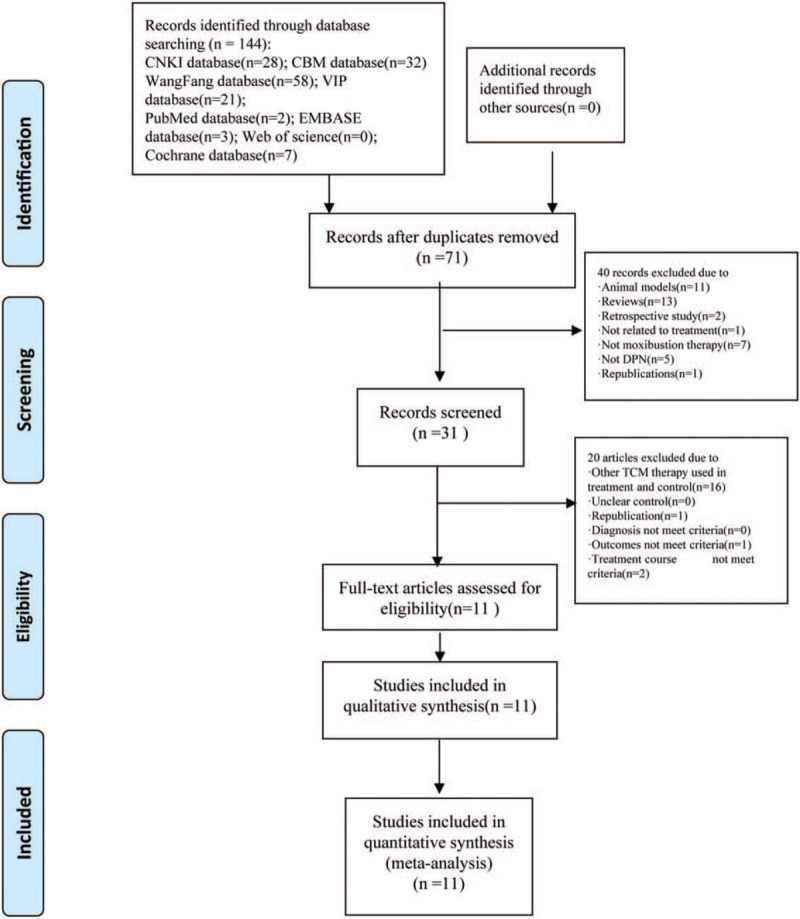
Flow chart of the literature screen.

As displayed in Figure 1, our search strategy initially identified a total of 144 records. After removing 73 duplicates, further 40 irrelevant records were excluded by screening the titles and abstracts. A full-text analysis of 31 potentially relevant articles excluded an additional 20 articles because other TCM therapies were used in treatment and control (n = 16), republications (n = 1), outcomes not meet criteria (n = 1), treatment course not meet criteria (n = 2), respectively. Finally, 11 RCTs met our eligibility criteria and were selected for the meta-analysis.^[15–25]^

### 3.2 Description of selected studies

All 11 included RCTs were conducted in China, 1 was published in English.^[22]^ The characteristics of the 11 studies are presented in Table 1. The sample size of individual studies varied from 39 to 132 participants. In 5 studies, enrolled patients suffered from type-2 diabetes,^[15,16,19,20,23]^ and the other studies enrolled patients with no restriction to the type of diabetes. All included patients met the diagnostic criteria for DPN. Additionally, 4 trials reported the TCM syndromes of their participants.^[19,20,22,25]^

**Table 1:**
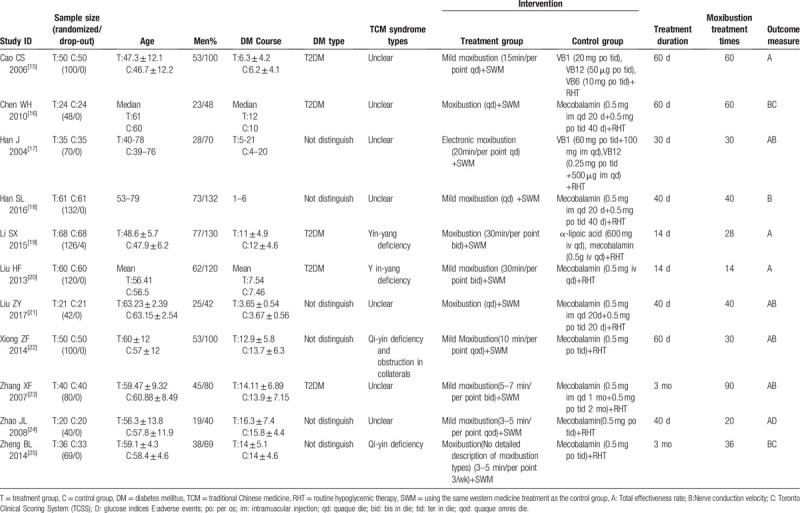
Characteristics of the included studies.

Participants in both groups received hypoglycemic therapy. All treatment groups received a combination of moxibustion plus WM therapies, and control groups received only WM interventions. Six trials used mild moxibustion (i.e., the lighted Moxa sticks were pointed to the acupoint for several minutes until flush and hot feelings were detected).^[15,18,20,22–24]^ In 1 trial,^[17]^ electronic moxibustion was used. One trial^[25]^ did not provide a clear description of the moxibustion equipment. Other trials^[16,19,21]^ were performed with moxibustion equipment (i.e., moxibustion-massage apparatus). WM treatments in most of the included trials used mecobalamin (8/11), other trials used vitamin B1, vitamin B12, vitamin B6, or α-lipoic acid. The studies’ treatment duration ranged from 14 days to 3 months. The moxibustion treatment was administered from 14 to 90 times. All the specific moxibustion acupoints adopted in 11 studies are shown in Table 2.

**Table 2:**
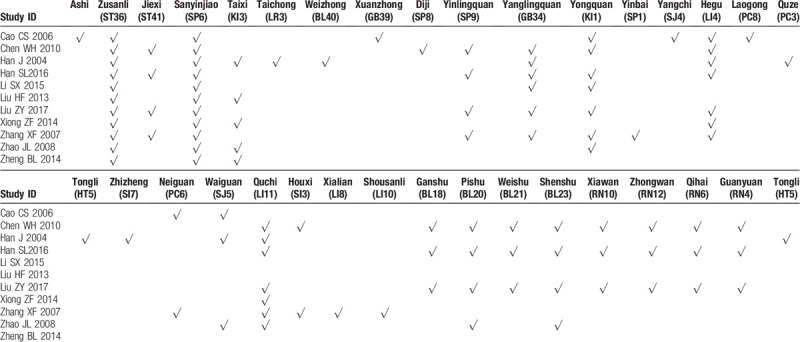
The specific moxibustion acupoints adopted in all 11 studies.

Seven trials reported SNCV and MNCV outcomes.^[16–18,21–23,25]^ Eight trials reported the total effectiveness rate that consisted of 2 parts: the subjective part (the symptoms and physical signs disappeared, improved, or alleviated) and the objective part (improvements of nerve-conduction velocity, tendon reflex, and deep sense). Finally, 2 trials reported the TCSS and only 1 trial reported glucose indices.^[16,24,25]^

### 3.3 Methodological quality within studies

**Figure 2:**
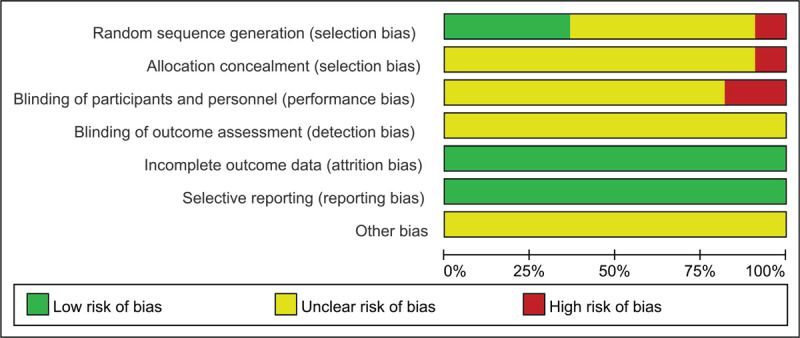
Risk of bias graph of included studies.

**Figure 3:**
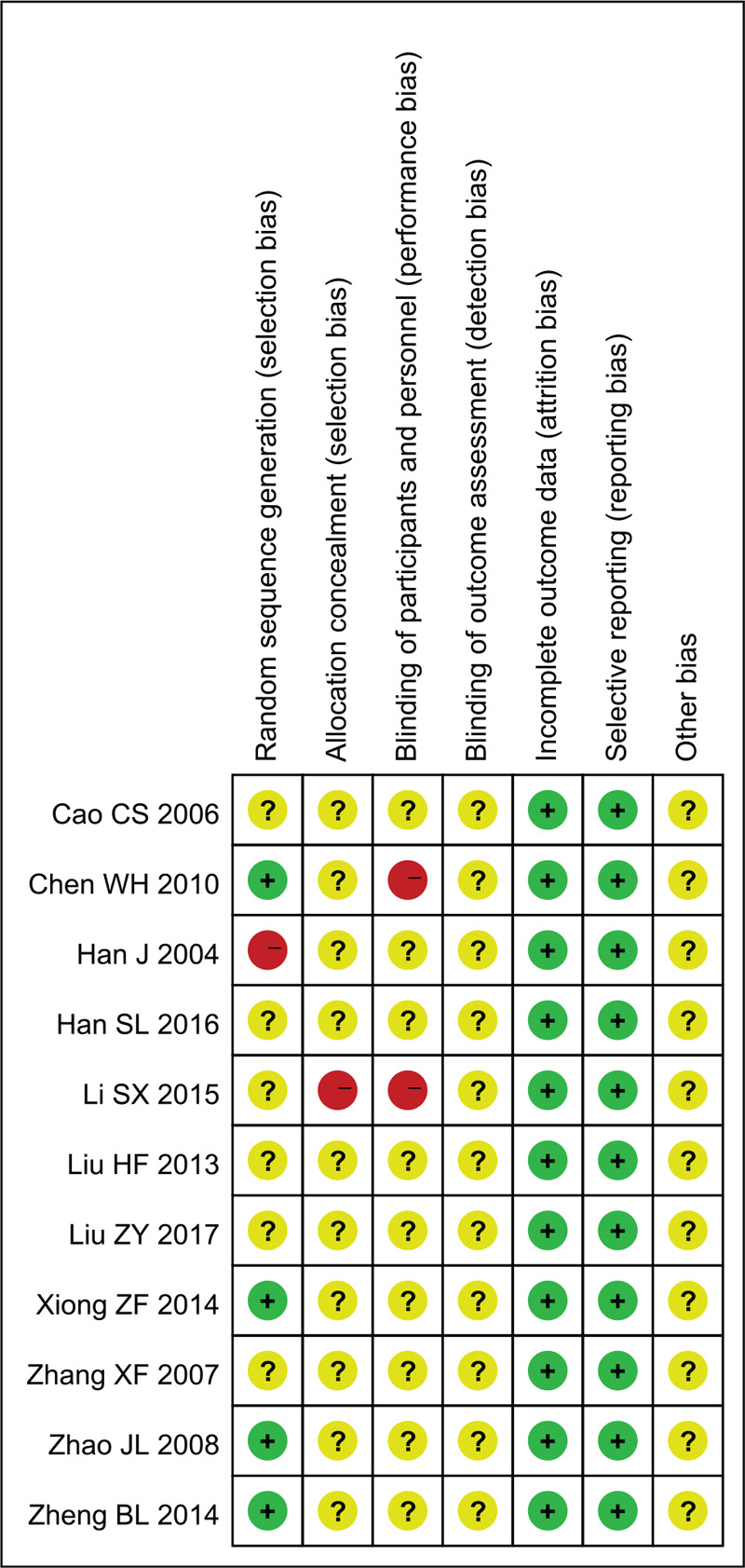
Risk of bias summary of included studies.

The results of the bias-risk assessment are shown in Figures 2 and 3. Of the 11 studies, 4 RCTs adopted strict randomization and reported their methods of random-number-sequence generation in detail. Two studies were assessed as a “high risk” for the random-number-sequence generation.^[17]^ Since insufficient information was available on the allocation concealment, blinding of participants and personnel, blinding of outcome assessment, and other bias in all 11 trials, the above items were judged to be “unclear.” There was no attrition bias in the 11 studies since the outcome data were complete. None of the 11 studies was found to have reporting bias.

### 3.4 Meta-analysis results

#### 3.4.1 Nerve-conduction velocity (NCV)

Seven trials reported outcomes of NCV and those that did, examined different nerves. Seven trials provided the MNCV and SNCV of the median nerve, 6 trials provided the MNCV and SNCV of the peroneal nerve.

##### 3.4.1.1 Median MNCV

**Figure 4:**
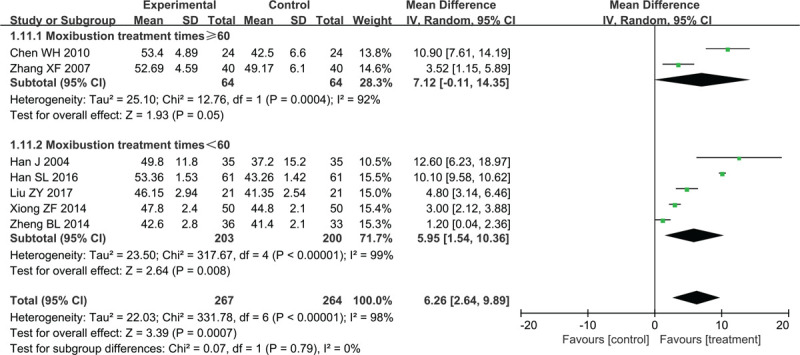
Forest plots of moxibustion effects on median MNCV. MNCV = motor nerve conduction velocity.

Seven trials assessed the changes of median MNCV with high heterogeneity between trials (*χ*^2^ = 331.78, *P* < .00001, I^2^ = 98%); a random-effect model was used for statistical analysis. Compared with control groups, treatment groups improved median MNCV significantly (MD = 6.26, 95% CI 2.64–9.89, Z = 3.39, *P* = .0007) (Fig. 4).

Anticipating that different moxibustion-treatment times may affect the results, subgroup analysis was performed. We divided studies into 2 subgroups according to different moxibustion-treatment times. Two studies with ≥60 moxibustion treatments reported median MNCV as an outcome (MD = 7.12, 95% CI: −0.11 to 14.35, Z = 1.93, *P* = .05); high heterogeneity between trials was observed (*χ*^2^ = 12.76, *P* = .0004, I^2^ = 92%). The treatment groups from 5 studies with <60 moxibustion treatments were superior to control groups in terms of median MNCV (MD = 5.95, 95% CI: 1.54–10.36, Z = 2.64, *P* = .008). These 5 studies also showed significant heterogeneity of the trial results (*χ*^2^ = 317.67, *P* < .00001, I^2^ = 99%).

##### 3.4.1.2 Peroneal MNCV

**Figure 5:**
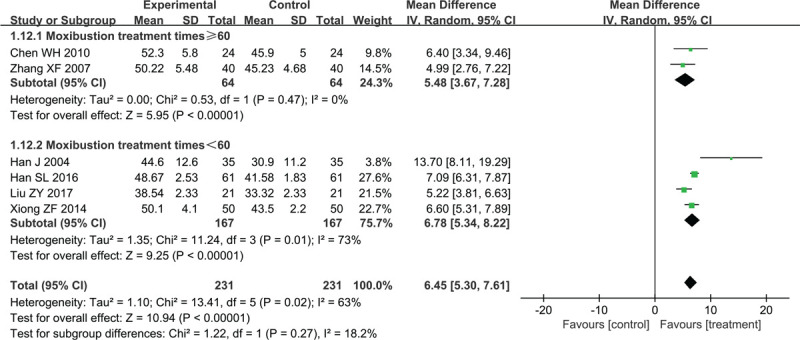
Forest plots of moxibustion effects on peroneal MNCV. MNCV = motor nerve conduction velocity.

As shown in Figure 5, 6 trials reported the changes in peroneal MNCV (MD = 6.45, 95% CI 5.30–7.61, *P* < .00001). We used the random-effect model because of the significant heterogeneity between trials (*χ*^2^ = 13.41, *P* = .02, I^2^ = 63%).

Two studies with moxibustion treatment times ≥60 compared the peroneal MNCV between treatment groups and control groups (MD = 5.48, 95% CI 3.37–7.28, Z = 5.95, *P* < .00001) without heterogeneity (*χ*^2^ = 0.53, *P* = .47, I^2^ = 0%). The other 4 trials used less than 60 moxibustion treatments and compared the peroneal MNCV between treatment groups and control groups (MD = 6.78, 95% CI 5.34–8.22, Z = 9.25, *P* < .00001) with high heterogeneity (*χ*^2^ = 11.24, *P* = .01, I^2^ = 73%). Sensitivity analysis showed that heterogeneity decreased after studies Han et al and Liu and Zhang were removed from the <60 moxibustion-treatments subgroup (*χ*^2^ = 0.41, *P* = .36, I^2^ = 0%). Therefore, we speculate that the possible sources of heterogeneity may be related to the type of moxibustion used and the degree of peripheral neuropathy.

##### 3.4.1.3 Median SNCV

**Figure 6:**
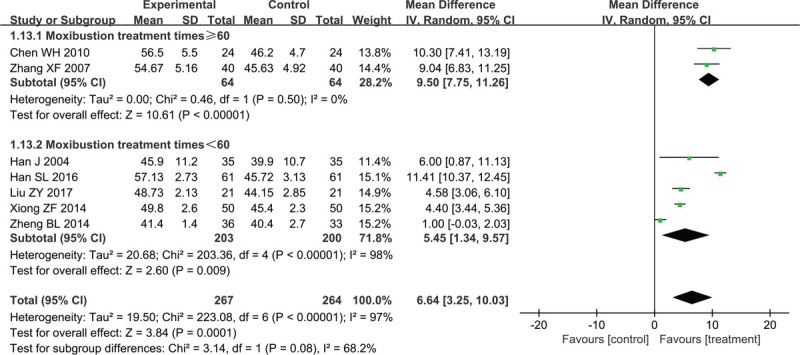
Forest plots of moxibustion effects on median SNCV. SNCV = sensory nerve conduction velocity.

Seven studies that involved 531 patients reported changes in median SNCV. Significant heterogeneity between trials was observed (*χ*^2^ = 223.08, *P* < .00001, I^2^ = 97%), a random-effect model was applied for statistical analysis. There was a significant increase in the median SNCV compared with the control group (MD = 6.64, 95% CI 3.25–10.03, *P* = .0001) (Fig. 6).

Two studies with ≥60 moxibustion treatments compared the median SNCV between treatment groups and control groups (MD = 9. 50, 95% CI 7.75–11.26, Z = 10.61, *P* < .00001) without heterogeneity (*χ*^2^ = 0.46, *P* = .5, I^2^ = 0%). The other 5 trials that had less than 60 moxibustion treatments compared the median SNCV between treatment groups and control groups (MD = 5.45, 95% CI 1.34–9.57, Z = 2.60, *P* = .0009) with high heterogeneity (*χ*^2^ = 203.36, *P* < .00001, I^2^ = 98%). Heterogeneity was reduced after studies by Han et al and Zheng et al were removed from the moxibustion-treatment times <60 subgroup (*χ*^2^ = 0.38, *P* = .83, I^2^ = 0%). The heterogeneity may have been caused by the moxibustion type used; the Han et al study used electronic moxibustion and the Zheng et al study did not describe in enough detail the type of moxibustion that was employed.

##### 3.4.1.4 Peroneal SNCV

**Figure 7:**
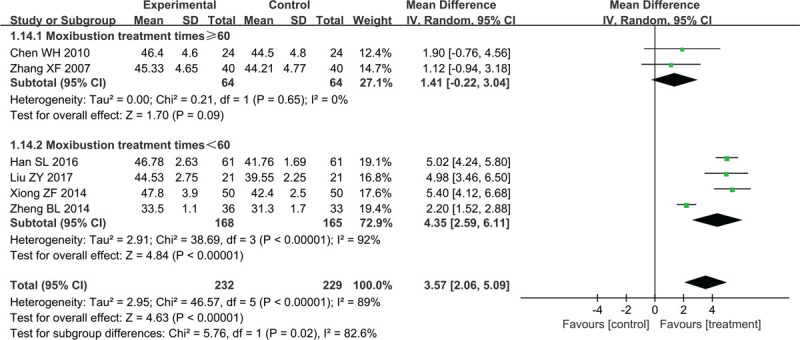
Forest plots of moxibustion effects on peroneal SNCV. SNCV = sensory nerve conduction velocity.

Six studies involving 461patients reported peroneal SNCV data (MD = 3. 57, 95% CI 2.06–5.09, Z = 4.63, *P* < .00001) (Fig. 7); a random-effect model was used because of the high heterogeneity between studies (*χ*^2^ = 46.57, *P* < .00001, I^2^ = 89%).

Two studies with ≥60 moxibustion treatments compared the peroneal SNCV between the treatment and control groups (MD = 1. 41, 95% CI −0.22 to 3.04, Z = 1.70, *P* = .09) without heterogeneity (*χ*^2^ = 0.21, *P* = .65, I^2^ = 0%). The other 4 trials that used less than 60 moxibustion treatments compared the peroneal SNCV between the treatment and control groups (MD = 4.35, 95% CI 2.59 to 6.11, Z = 4.84, *P* < .00001) with high heterogeneity (*χ*^2^ = 38.69, *P* < .00001, I^2^ = 92%). Heterogeneity was reduced after the Zheng et al study was removed from the <60 moxibustion treatments subgroup (*χ*^2^ = 0.27, *P* = .87, I^2^ = 0%). The lack of specific details about the instrument and the type of moxibustion treatment used in the Zheng et al study may be the reason for the observed heterogeneity.

#### 3.4.2 Total effectiveness rate

**Figure 8:**
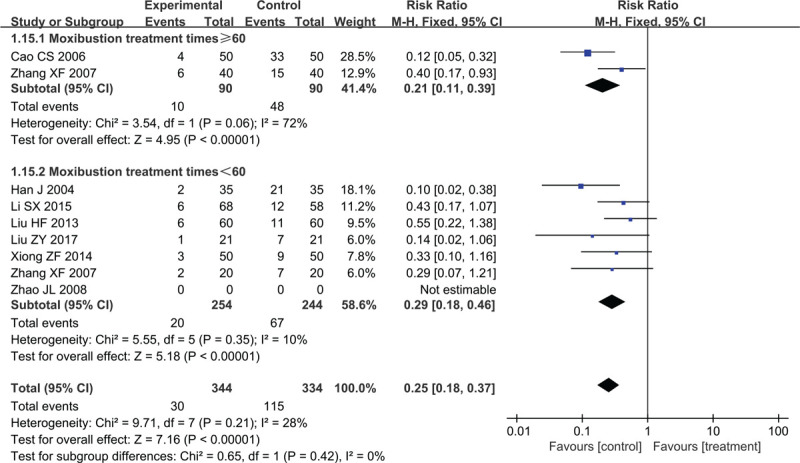
Forest plots of moxibustion effects on total effectiveness rate.

Eight studies involving 678 participants reported the total-effectiveness-rate outcome. Due to the low heterogeneity (*χ*^2^ = 9.71, *P* = .21, I^2^ = 28%) (Fig. 8), we used a fixed-effect model for the combined analysis. The treatment groups indicated a better total-effectiveness rate than the control groups (RR = 0.25, 95% CI 0.18–0.37, Z = 7.16, *P* < .00001).

In subgroup analysis, the total effective rate of the treatment group was higher than that of the control group, with the ≥60 moxibustion-treatment group showing high heterogeneity (*χ*^2^ = 3.54, *P* = .06, I^2^ = 72%; RR = 0.21, 95% CI 0.11–0.39, Z = 4.95, *P* < .00001) and the <60 moxibustion-treatment group showing low heterogeneity (*χ*^2^ = 5.55, *P* = .35, I^2^ = 10%; RR = 0.29, 95% CI 0.18–0.46, Z = 5.18, *P* < .00001).

#### 3.4.3 Toronto clinical scoring system (TCSS)

**Figure 9:**
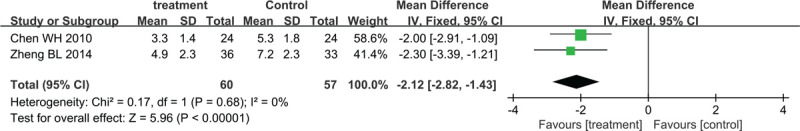
Forest plots of moxibustion effects on TCSS. TCSS = Toronto Clinical Scoring System.

As shown in Figure 9, 2 studies investigated TCSS. Based on the low heterogeneity, the meta-analysis was performed using a fixed-effect model (*χ*^2^ = 0.17, *P* = .68, I^2^ = 0%). Compared with the control groups, TCSS indicated a significant decrease in the treatment groups (MD = −2.12, 95% CI −2.82 to 1.43, *P* < .00001).

#### 3.4.4 Adverse events

Only 1 study mentioned adverse events,^[16]^ however, there were no serious side effects reported for the treatment period in the treatment and control groups. The other 10 studies did not mention adverse events.

### 3.5 Publication bias

**Figure 10:**
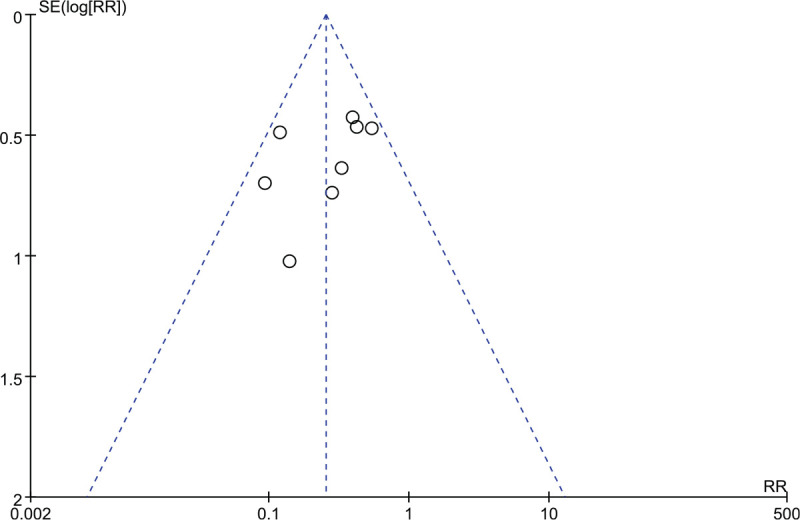
Funnel plot for assessing publication bias.

The outcomes of the total-effectiveness rate involving 8 studies were tested for publication bias. As indicated in Figure 10, the funnel-graph shape was visually imperfectly symmetrical indicating a potential publication bias.

## 4 Discussion

DPN is the most common complication of diabetes that may lead to the occurrence of diabetic foot ulcers and even to foot or limb amputations. At present, the main important strategy to prevent and treat DPN is to control hyperglycemia and keep the blood-glucose level stable. Other therapeutic approaches to control DPN including those evaluated in clinical trials have shown limited efficacy especially on painless symptoms.^[9,26]^

According to the theory of TCM, DPN belongs to the “bi syndrome” of TCM and is related to the blockage of meridians. Moxibustion can warm Yang, eliminate cold, and dredge meridians, and is a representative external treatment in TCM. Based on meridians and acupoints, it can intervene in various diseases utilizing heat, light radiation, and drug effects.^[27]^ Clinical effectiveness of moxibustion in DPN has been widely recognized. A study demonstrated that moxibustion could improve DPN-related neuroinflammation by restoring the balance between NF-κB and Nrf2 in rats and may thus be complementary to the current treatment of DPN.^[9]^ Our study evaluated the efficacy and safety of moxibustion in treating DPN.

The study conducted a meta-analysis of 11 studies involving 927 patients. The data suggested that the efficacy of moxibustion in treating DPN was significantly better than that of the control groups. NCV is considered to be the most objective and reliable method in the diagnosis of DPN having 40% sensitivity and 100% specificity ^[28]^ and was the primary outcome criterion in our analysis; however, only 7 studies reported NCV results and the nerves examined by NCV were not identical. Therefore, it was necessary to standardize NCV evaluation to improve the reliability of data analysis. Moreover, we speculated that many factors would influence the results performed by subgroup and sensitivity analyses such as moxibustion type, the times of moxibustion treatment, the degree of peripheral neuropathy, and so on. Such factors will need to be controlled in future clinical trials involving moxibustion applied to DPN treatment. Only 2 studies reported outcomes based on the assessment of TCSS symptoms. TCSS is a relatively simple, comprehensive, and effective screening method that includes symptoms and signs. The examination is relatively objective, consistent with clinical examinations, and highly reliable.^[29]^ Accordingly, it is essential to standardize the assessment of symptoms.

Only 1 study reported changes in blood-glucose level,^[24]^ hence we could not evaluate the effect of moxibustion treatment on this parameter. Consequently, it remains unclear whether the improvement of outcomes such as NVC is related to an improvement of blood-glucose levels. Two studies have shown that moxibustion can improve hemorheological indexes^[17,24]^; this effect may also be related to improvements in neurological function. More pharmacological and clinical studies are still required to verify the mechanism of moxibustion in treating DPN.

**Figure 11:**
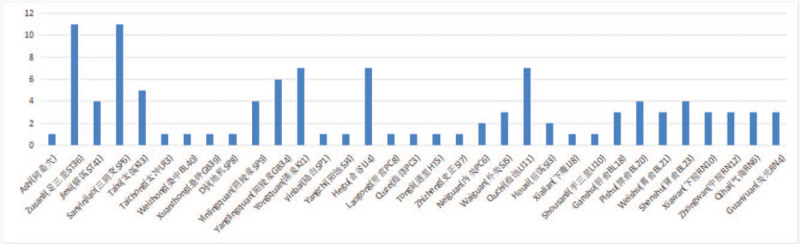
Acupoints used in the included trials.

All the 11 trials reported specific acupoints applied in their treatment; this data might provide a reference for acupoints selection in clinical practice. The 5 most frequently used acupoints were Zusanli (ST36), Sanyinjiao (SP6), Yanglingquan (GB34), Hegu (LI4), and Quchi (LI11). We also found that most of the acupoints used in the 11 trials are located on limbs, with some being located at the abdomen (e.g., Zhongwan (RN12), Xiawan (RN10), Qihai (RN6), etc.) and back (e.g., Pishu (BL20), Weishu (BL21), Shenshu (BL23), etc.) (Fig. 11). Acupoints have not only local effects but also systemic effects based on their meridian route.

This meta-analysis has several limitations. All the clinical studies included in our analysis were performed in China; this may suggest that the reported positive results might have a likelihood of publication bias possibly resulting from high heterogeneity, an insufficient number of trials, and a small sample size. Further, no follow-up study and long-term effects of moxibustion on DPN were reported. Additionally, the use of different therapeutic acupoints, treatment frequency, and moxibustion equipment would likely affect the result; we were not able to assess this due to the lack of such data in the included studies.

Although the conclusion of our meta-analysis is limited, it may still provide some inspiration. For any follow-up study, establishing methodological quality is critical. For example, the studies we analyzed generally failed to ensure patient blinding; suitable devices for sham moxibustion treatment will be necessary for future studies. Moreover, attention must be paid to adverse events because moxibustion is not free of risks and generates heat, smoke, and tar that may present a risk of adverse events. The availability of a large amount of safety data will be necessary to standardize the moxibustion therapy.

## 5 Conclusions

Moxibustion therapy has been shown to have better clinical effects compared with control treatments and to be an effective and safe alternative for treating DPN patients. However, due to the poor methodological quality of the included trials, more rigorous RCTs are required to evaluate the efficacy and safety of moxibustion before definitive recommendations for the use of the procedure to treat DPN patients can be made.

## Author contributions

**Investigation:** Qian Wu, Yi Zhang, Qing Pang, Meizhen Zhang, Yanan Yang

**Methodology:** Bing Pang, Lijuan Du, Qing Ni

**Writing – original draft:** Yumeng Tan, Jun Hu

**Writing – review & editing:** Yumeng Tan

## Supplementary Material

Supplemental Digital Content
