# Supplementary material for: Moxibustion for the treatment of diabetic peripheral neuropathy: A systematic review and meta-analysis following PRISMA guidelines
Source: Medicine (Baltimore). 2020 Sep 25;99(39):e22286. doi: 10.1097/MD.0000000000022286 (PMC7523832; doi:10.1097/MD.0000000000022286)
Supplement: Supplemental Digital Content [file medi-99-e22286-s001.pdf]

Moxibustion for the treatment of diabetic peripheral neuropathy: a systematic review and meta-analysis following PRISMA guidelines

Relevant studies will be obtained from eight electronic databases, including Cochrane Library, PubMed, EMBASE, Web of Science, Chinese National Knowledge Infrastructure database (CNKI), Chinese Biomedical Database (CBM), Chinese Science and Technique Journal Database (VIP), and Wan Fang Database. The search will be performed in English and Chinese.

Search Strategy for PubMed

#1 diabetic neuropathy[mh]
#2 diabetic peripheral neuropathy[all fields]
#3 diabetic neuropathies[all fields]
#4 DPN [all fields]
#5 #1 OR #2 OR #3 OR #4
#6 moxibustion[mh]
#7 moxa[all fields]
#8 moxa-moxibustion[all fields]
#9 warm-moxibustion[all fields]
#10 mild-moxibustion[all fields]
#11 indirect-moxibustion[all fields]
#12 #6 OR #7 OR #8 OR #9 OR #10 OR #11
#13 #5 AND #12
#14 randomized controlled trial[pt]
#15 controlled clinical trial[pt]
#16 randomized[tiab]
#17 placebo[tiab]
#18 randomly[tiab]
#19 #14 OR #15 OR #16 OR #17 OR #18
#20 #13 AND #19
